# A five-compartment model of age-specific transmissibility of SARS-CoV-2

**DOI:** 10.1186/s40249-020-00735-x

**Published:** 2020-08-26

**Authors:** Ze-Yu Zhao, Yuan-Zhao Zhu, Jing-Wen Xu, Shi-Xiong Hu, Qing-Qing Hu, Zhao Lei, Jia Rui, Xing-Chun Liu, Yao Wang, Meng Yang, Li Luo, Shan-Shan Yu, Jia Li, Ruo-Yun Liu, Fang Xie, Ying-Ying Su, Yi-Chen Chiang, Ben-Hua Zhao, Jing-An Cui, Ling Yin, Yan-Hua Su, Qing-Long Zhao, Li-Dong Gao, Tian-Mu Chen

**Affiliations:** 1 State Key Laboratory of Molecular Vaccinology and Molecular Diagnostics, School of Public Health, Xiamen University, 4221-117 South Xiang’an Road, Xiang’an District, Xiamen City, 361102 Fujian Province People’s Republic of China; 2 Hunan Provincial Center for Disease Control and Prevention, 405 Furong Middle Road Section One, Kaifu District, Changsha City, 410001 Hunan Province People’s Republic of China; 3 Division of Public Health, School of Medicine, University of Utah, 201 Presidents Circle, Salt Lake City, UT 84112 USA; 4 Department of Mathematics, School of Science, Beijing University of Civil Engineering and Architecture, Beijing, People’s Republic of China; 5 Shenzhen Institutes of Advanced Technology, Chinese Academy of Sciences, Shenzhen, Guangdong Province People’s Republic of China; 6 Jilin Provincial Center for Disease Control and Prevention, 3145 Jingyang Big Road, Lvyuan District, Changchun, 130062 Jilin Province People’s Republic of China

**Keywords:** Transmissibility, SARS-CoV-2, COVID-19, Compartmental model, Age-specific dynamics

## Abstract

**Background:**

The novel coronavirus, severe acute respiratory syndrome coronavirus 2 (SARS-CoV-2, also called 2019-nCoV) causes different morbidity risks to individuals in different age groups. This study attempts to quantify the age-specific transmissibility using a mathematical model.

**Methods:**

An epidemiological model with five compartments (susceptible–exposed–symptomatic–asymptomatic–recovered/removed [SEIAR]) was developed based on observed transmission features. Coronavirus disease 2019 (COVID-19) cases were divided into four age groups: group 1, those ≤ 14 years old; group 2, those 15 to 44 years old; group 3, those 45 to 64 years old; and group 4, those ≥ 65 years old. The model was initially based on cases (including imported cases and secondary cases) collected in Hunan Province from January 5 to February 19, 2020. Another dataset, from Jilin Province, was used to test the model.

**Results:**

The age-specific SEIAR model fitted the data well in each age group (*P* < 0.001). In Hunan Province, the highest transmissibility was from age group 4 to 3 (median: *β*_43_ = 7.71 × 10^− 9^; *SAR*_43_ = 3.86 × 10^− 8^), followed by group 3 to 4 (median: *β*_34_ = 3.07 × 10^− 9^; *SAR*_34_ = 1.53 × 10^− 8^), group 2 to 2 (median: *β*_22_ = 1.24 × 10^− 9^; *SAR*_22_ = 6.21 × 10^− 9^), and group 3 to 1 (median: *β*_31_ = 4.10 × 10^− 10^; *SAR*_31_ = 2.08 × 10^− 9^). The lowest transmissibility was from age group 3 to 3 (median: *β*_33_ = 1.64 × 10^− 19^; *SAR*_33_ = 8.19 × 10^− 19^), followed by group 4 to 4 (median: *β*_44_ = 3.66 × 10^− 17^; *SAR*_44_ = 1.83 × 10^− 16^), group 3 to 2 (median: *β*_32_ = 1.21 × 10^− 16^; *SAR*_32_ = 6.06 × 10^− 16^), and group 1 to 4 (median: *β*_14_ = 7.20 × 10^− 14^; *SAR*_14_ = 3.60 × 10^− 13^). In Jilin Province, the highest transmissibility occurred from age group 4 to 4 (median: *β*_43_ = 4.27 × 10^− 8^; *SAR*_43_ = 2.13 × 10^− 7^), followed by group 3 to 4 (median: *β*_34_ = 1.81 × 10^− 8^; *SAR*_34_ = 9.03 × 10^− 8^).

**Conclusions:**

SARS-CoV-2 exhibits high transmissibility between middle-aged (45 to 64 years old) and elderly (≥ 65 years old) people. Children (≤ 14 years old) have very low susceptibility to COVID-19. This study will improve our understanding of the transmission feature of SARS-CoV-2 in different age groups and suggest the most prevention measures should be applied to middle-aged and elderly people.

## Background

Coronavirus disease 2019 (COVID-19), caused by severe acute respiratory syndrome coronavirus 2 (SARS-CoV-2, also called 2019-nCoV), has spread around the world. It has been evident from the start of the pandemic that different age groups are at different risks for COVID-19. During the earliest stage of the spread (before January 2, 2020), it was noticed that most transmission involved persons over 18 years old, especially persons aged 25 to 64 [1]. For example, a study involving 425 COVID-19 patients found that they ranged in age from 15 to 89, with a median of 59. Different case distributions were reported in four age groups: 0 to 14, 15 to 44, 45 to 64, and ≥ 65 years [2]. In China, persons 30 to 79 years old accounted for 86.6% of diagnosed cases [3]. The distribution of cases in the Republic of Korea was mainly concentrated in the range of 20–50 years; the peak age was 30 [4]. It has been reported that older people exhibit a high risk of developing severe symptoms or even dying [5]. However, another study indicates that children and adults face the same infectious risk [6], although there have also been reports that younger adults, especially those aged 20 to 24 years, face an increasing risk of COVID-19 after an intervention [7]. Research has also shown that individuals over 65 years of age are more susceptible to infection than those 14 to 64 years old (odds ratio: 1.47, 95% *CI*: 1.12–1.92) [8]. Looking at these diverse results of age characteristics, it is clear that the role of age in transmission needs to be clarified. In this study, interpersonal transmission of COVID-19 will be further explored to provide improved estimation of transmissibility at different ages.

Several approaches to the mathematical modeling of COVID-19 [9, 10], such as calculating the basic reproduction number (*R*_0_) using the serial intervals and intrinsic growth rate [2, 9, 10], or using ordinary differential equations and Markov chain Monte Carlo methods [11], have been proposed. Compartmental models are often applied to infectious diseases, and have sometimes been used to study age-dependent effects [12, 13]. For example, Chen et al. developed a Bats–Hosts–Reservoir–People (BHRP) transmission network model and simplified the BHRP model as a person–person (PP) transmission network model to calculate the transmissibility of SARS-CoV-2 [12]. The age-specific transmissibility of influenza A (H1N1) has been studied in a model with five compartments (the Susceptible–Exposed–Symptomatic–Asymptomatic–Recovered/removed [SEIAR] model) [13]. However, no such compartmental model is available for quantifying the age-specific transmissibility of SARS-CoV-2.

In this paper an age-specific SEIAR model based on the PP model is proposed. It is employed to estimate the age-specific transmissibility of SARS-CoV-2 by fitting data collected in Hunan province between January 5 and February 19, 2020. A dataset of COVID-19 cases from Jilin Province is used to test the model further.

## Methods

### Data collection

The present model is based on COVID-19 cases data collected by the Hunan Provincial Center for Disease Control and Prevention (Hunan Provincial CDC) from January 5 to February 19, 2020. The data included patient gender, age, inter-provincial travel history, case type (symptomatic/asymptomatic), exposure date, date of onset, and date of diagnosis. To further test the model, a separate dataset (including age, travel history, case type, and date of onset) collected in Jilin Province from January 5 to February 12, 2020 was also used.

### Study design

In this study, COVID-19 patients were divided into four age groups, as is done elsewhere in the published literature [2]. Age-group 1 contained those people who were ≤ 14 years old, group 2 aged 15 to 44 years old, group 3 with those 45 to 64 years old, and group 4 of those ≥ 65 years old. Moreover, each age group was divided into two types, including imported cases (patients who had traveled from other provinces) and secondary cases (patients infected within their home province by imported and local cases). All cases were classified as symptomatic or asymptomatic.

### Age-specific transmission model

The age-specific SEIAR model is based on the natural history of COVID-19. In the model, people are sorted into five compartments (categories): susceptible (*S*), exposed (*E*), symptomatic (*I*), asymptomatic (*A*), and recovered/removed (*R*). The definitions of the five categories are presented in Table 1. The model is based on the following assumptions:

- Susceptible individuals infected by contact with two types of cases: symptomatic/asymptomatic cases from other provinces and secondary cases in their home province. The imported symptomatic individuals are placed in the subcategory *I*_*p*_ and the imported asymptomatic individuals in the subcategory *A*_*p*_.
- SARS-CoV-2 can be transmitted within each age group. The transmission rate within a given age group *i* is denoted as *β*_*ii*_.
- SARS-CoV-2 can be transmitted between different age groups. The transmission rate from age group *i* to *j* is *β*_*ij*_ and that from *j* to *i* is *β*_*ji*_.
- The incubation period of an exposed person is 1/*ω*, the latent period by 1/*ω*’. The model assumes that the incubation period is equal to the latent period. Parameter *p* (0 ≤ *p* ≤ 1) gives the proportion of individuals who are asymptomatically infected. Exposed individuals move out of the *E* compartment into the *A* compartment at a rate of *pωE* and into the *I* (symptomatic) compartment at a rate of (1*-p*)*ωE*.
- The transmissibility of the virus from members of *A* and that from members of *I* differ by a factor *κ* (0 ≤ *κ* ≤ 1).
- The model assumes that infected individuals only spread the virus until they are diagnosed, because (whether symptomatic or asymptomatic) they are removed from the population immediately upon diagnosis. More formally, individuals in categories *I* and *A* are transferred into category *R* after an infectious period of 1/*γ* and 1/*γ*’, respectively. Moreover, some members of *I* will die as a result of the infection. The case fatality rate is denoted *f*.

**Table 1:** Variables in the age-specific SEIAR model

Variables	Description	Unit
S_*i*_	Susceptible individuals density of age group *i*	Individuals·km^−2^
S_*j*_	Susceptible individuals density of age group *j*	Individuals·km^−2^
E_*i*_	Exposed individuals density of age group *i*	Individuals·km^−2^
E_*j*_	Exposed individuals density of age group *j*	Individuals·km^−2^
I_*i*_	Infectious individuals density age group *i*	Individuals·km^−2^
I_*j*_	Infectious individuals density age group *j*	Individuals·km^−2^
A_*i*_	Asymptomatic individuals density age group *i*	Individuals·km^−2^
A_*j*_	Asymptomatic individuals density age group *j*	Individuals·km^−2^
R_*i*_	Recovered/Removed individuals density age group *i*	Individuals·km^−2^
R_*j*_	Recovered/Removed individuals density age group *j*	Individuals·km^−2^
N	Total number of population density	Individuals·km^−2^

**Fig. 1:**
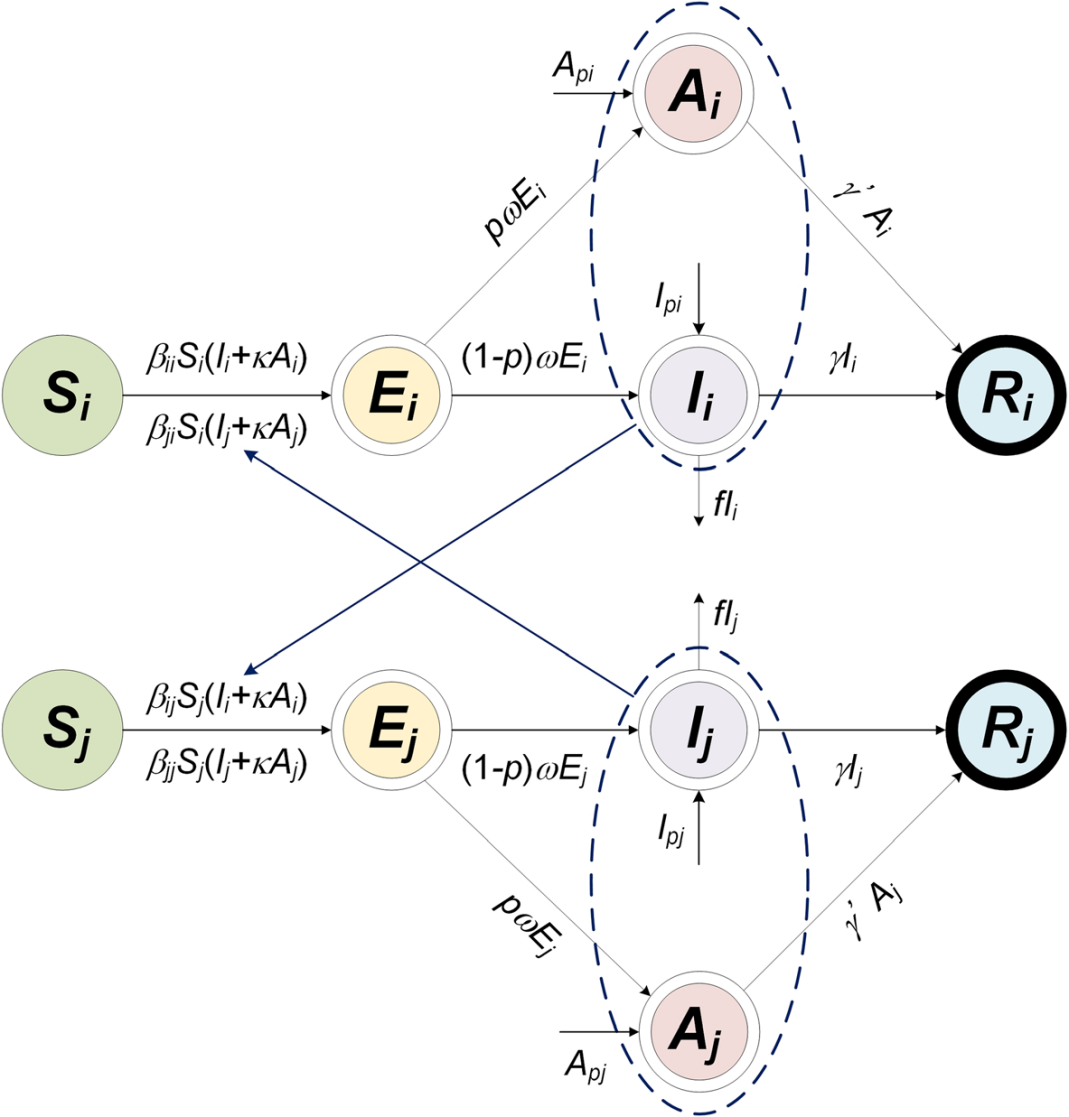
Flowchart of the age-specific SEIAR model. *i* and *j* represent age ≤ 14, 15–44, 45–64, and ≥ 65, respectively (*i* ≠ *j*)

A flowchart of the model is presented in Fig. 1. The equations of the age-specific SEIAR model are

$$ i\ne j $$

$$ \frac{d{S}_i}{dt}=-{\beta}_{ii}{S}_i\left({I}_i+\kappa {A}_i\right)-{\beta}_{ji}{S}_i\left({I}_j+\kappa {A}_j\right) $$

$$ \frac{d{E}_i}{dt}={\beta}_{ii}{S}_i\left({I}_i+\kappa {A}_i\right)+{\beta}_{ji}{S}_i\left({I}_j+\kappa {A}_j\right)-\omega {E}_i $$

$$ \frac{d{I}_i}{dt}={I}_{pi}+\left(1-p\right)\omega {E}_i-\gamma {I}_i-f{I}_i $$

$$ \frac{d{A}_i}{dt}={A}_{pi}+ p\omega {E}_i-{\gamma}^{\prime }{A}_i $$

$$ \frac{d{R}_i}{dt}={\gamma I}_i+{\gamma}^{\prime }{A}_i $$

$$ \frac{d{S}_j}{dt}=-{\beta}_{jj}{S}_j\left({I}_j+\kappa {A}_j\right)-{\beta}_{ij}{S}_j\left({I}_i+\kappa {A}_i\right) $$

$$ \frac{d{E}_j}{dt}={\beta}_{jj}{S}_j\left({I}_j+\kappa {A}_j\right)+{\beta}_{ij}{S}_j\left({I}_i+\kappa {A}_i\right)-\omega {E}_j $$

$$ \frac{d{I}_j}{dt}={I}_{pj}+\left(1-p\right)\omega {E}_j-\gamma {I}_j-f{I}_j $$

$$ \frac{d{A}_j}{dt}={A}_{pj}+ p\omega {E}_j-{\gamma}^{\prime }{A}_j $$

$$ \frac{d{R}_j}{dt}={\gamma I}_j+{\gamma}^{\prime }{A}_j $$

$$ N={S}_i+{E}_i+{I}_i+{A}_i+{R}_i $$

The *N* is defined as total population. The left side of the equation indicates the instantaneous change rate of *S*, *E*, *I*, *A*, and *R* at time *t*. The subscripts *i* and *j* (*i* ≠ *j*) represent age groups 1 to 4 in the respective equations.

### Parameter estimation

**Fig. 2:**
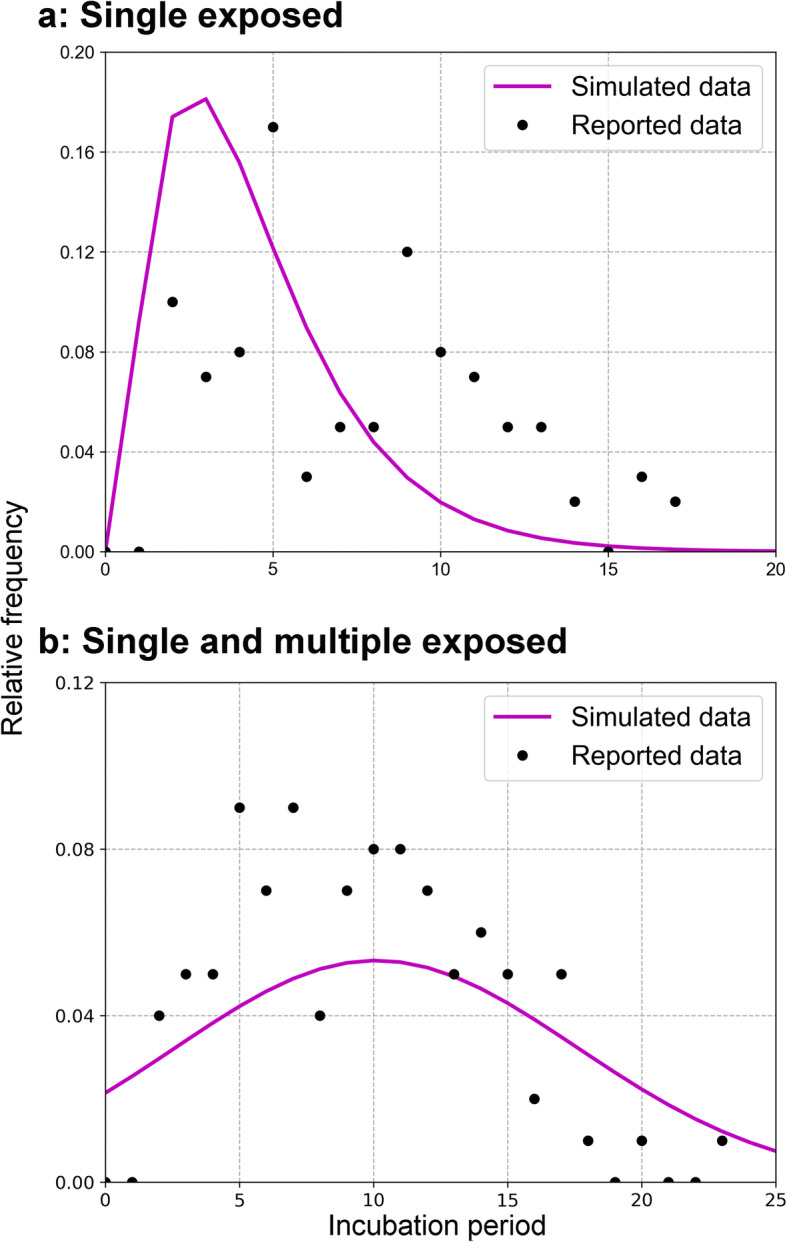
The fitting results of gamma distribution of incubation period. **a** Single exposed incubation period; **b** Single and multiple exposed incubation period

**Fig. 3:**
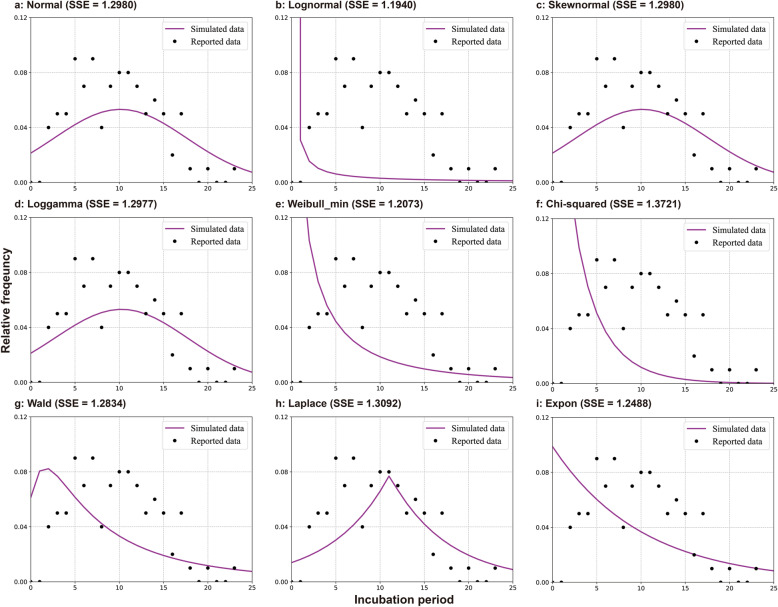
The fitting results of nine distribution of incubation period. **a** Normal; **b** Lognormal; **c** Skew-normal; **d** Log-gamma; **e** Weibull minimum; **f** Chi-square; **g** Wald; **h** Laplace; **i** Exponential

According to the literature, the incubation period was 4 days (interquartile range: 2–7) in the early epidemic in Wuhan City [14]; it was 5.1 days (95% *CI*: 4.5–5.8) according to other publicly reported data from China [15]. The incubation found by a survey in Ningbo City was 5.5 days (range: 2–18); the analysis of right truncation data in Wuhan City showed that the incubation period was 5 days (95% *CI*: 2–14) [16, 17]. However, the latent period has been reported much less often. In this study, a fit of first-hand data from Hubei Province (Additional file 1) using the gamma distribution gave an incubation period of 3 to 4 days for a single exposure (Fig. 2a) and 10 days for single and multiple exposures (Fig. 2b). Fits were also obtained with nine other distributions (normal, lognormal, skew-normal, log-gamma, Weibull minimum, chi-square, Wald, Laplace, and exponential); the normal, lognormal, and skew-normal distributions provided good fits similar to those with the gamma distribution (Fig. 3). In the model, the incubation period was set to 7 days, the average of the single and multiple exposure gamma-function results. Recalling our assumption that the incubation and latent periods are equal, *ω* = *ω’* = 0.1429, with a range from 0.05556–0.5.

According to reference [18], asymptomatic cases constitute 5 to 28% of all COVID-19 cases. The asymptomatic proportion in the Diamond Princess cruise ship was 17.9% (95% *CI*: 15.5–20.2%) [19]. One study adopted the binomial distribution to estimate the asymptomatic ratio as 30.8% (95% *CI*: 7.7–53.8%) [20]. The asymptomatic proportion was 20.75% in Ningbo City (15.8% among children) [16]. However, another study has indicated that the percentage of asymptomatic cases is much higher (78%) [21]. In the present work, first-hand data were used: according to the reported data in Hunan Province, 392 secondary cases included 79 asymptomatic ones. Therefore, the asymptomatic proportion (*p*) was set to 79/392 = 0.2015 in the present model.

One study has indicated that the spreading capacity of symptomatic cases is 3.9 times that of asymptomatic cases [22]. Another study indicated that individuals who closely contacted asymptomatic individuals infected after close contact with asymptomatic cases accounted for 4.11%, versus 6.30% for individuals infected after close contact with symptomatic cases [16]. According to a report from the UK [23], an asymptomatic individual may cause 11 infectious cases. In the model, *κ* is set to 1.0, thus conservatively allowing for the worst-case scenario that asymptomatic and symptomatic persons are equally infectious.

**Fig. 4:**
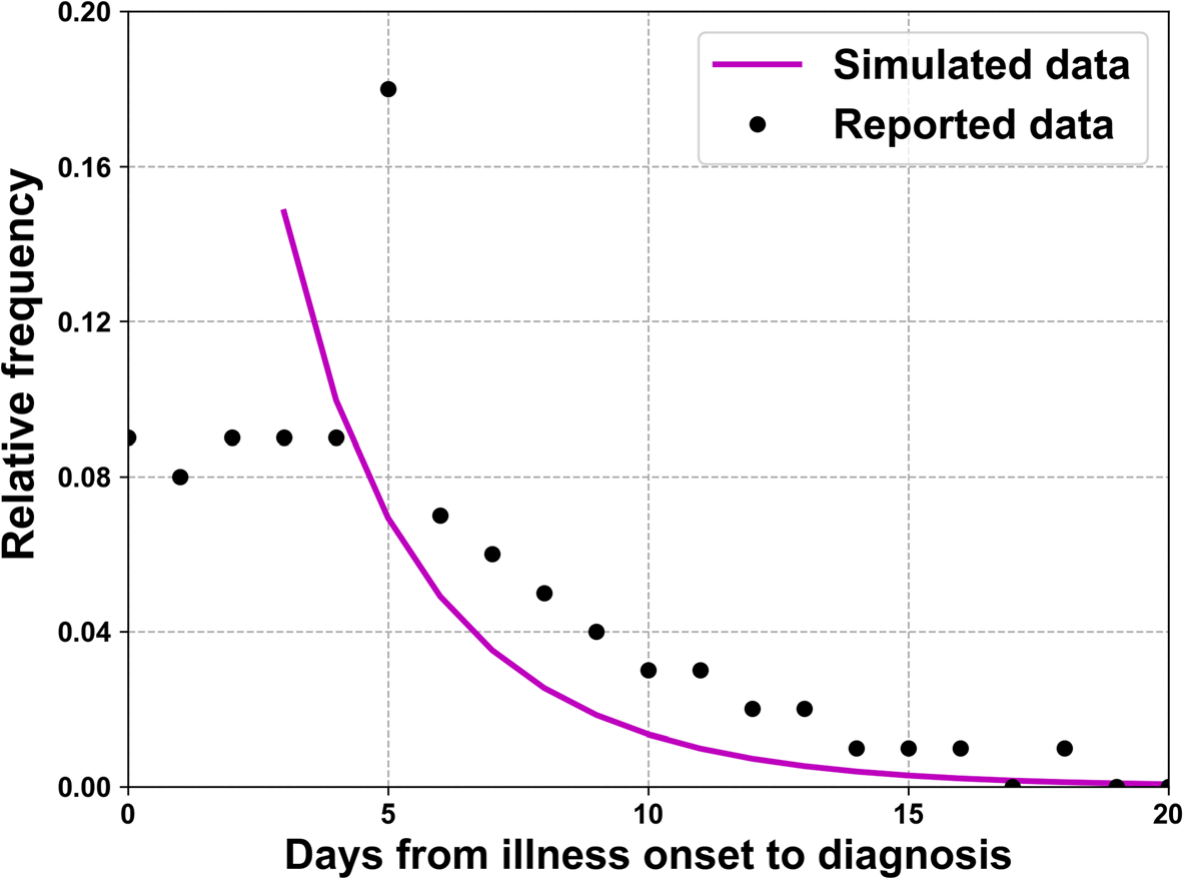
The fitting results of chi-square distribution of days from illness onset to diagnosis

A mean delay of 5 days has been reported from symptom onset to detection/hospitalization; in Thailand and Japan, patients were hospitalized between 3 and 7 days following onset [24–26]. Another study has indicated that the mean time from illness onset to hospital admission (for treatment and/or isolation) is 3 to 4 days without truncation and 5 to 9 days when right truncated [17]. However Xu et al. [27] reported that the median time from illness onset to initial hospital admission was 2 (range: 1–4) days. A study including 45 patients diagnosed prior to January 1, 2020 estimated the mean time from illness onset to first medical visit as 5.8 days (95% *CI*: 4.3–7.5) [2]. Another study indicated that the median communicable period of 24 asymptomatic cases was 9.5 days (range: 1–21) [28]. In this study, it is assumed that any person diagnosed with COVID-19 will be removed from the population immediately. Therefore, the infectious period is the same as the number of days from illness onset to diagnosis. Chi-square distribution results indicated that the highest frequency corresponded to day 5 (Fig. 4); therefore, the infectious period of the symptomatic and asymptomatic cases was set to 5 days in this study (*γ* = *γ’* = 0.2).

According to the analyses of the data collected by Hubei Provincial CDC, a total of four cases died as a result of COVID-19. Thus, the fatality rate (*f*) was set to 0.003552 (Table 2). In Hunan Province, the total population was set to 68 988 303 (≤ 14 years: 13 618 898; 15 to 44 years: 26 623 844; 45 to 64 years: 20 035 661; ≥ 65 years: 8 709 900). In Jilin Province, the same values as in Hunan Province were assumed for the parameters *κ*, *p*, *ω*, *γ*, *γ*’ and *f*, but the total population was set as 27 039 958 (≤ 14 years: 3 291 955; 15 to 44 years: 10 827 458; 45 to 64 years: 9 525 131; ≥ 65 years: 3 395 414).

**Table 2:** Description and values of parameters in the age-specific SEIAR model

Parameter	Description	Unit	Value	Range	Method
*β*_*ii*_^a^	Transmission relative rate among age group *i*	Individuals^− 1^·days^− 1^	–	≥ 0	Curve fitting
*β*_*ij*_^a^	Transmission relative rate from age group *i* to *j*	Individuals^−1^·days^− 1^	–	≥ 0	Curve fitting
*β*_*ji*_^a^	Transmission relative rate from age group *j* to *i*	Individuals^−1^·days^− 1^	–	≥ 0	Curve fitting
*β*_*jj*_^a^	Transmission relative rate among age group *i*	Individuals^−1^·days^− 1^	–	≥ 0	Curve fitting
*κ*	Relative transmissibility rate of asymptomatic to symptomatic individuals	1	1	0–1	Reference [16, 22, 23]
*p*	Proportion of the asymptomatic	1	0.2015	0.016–0.78	Analysis of data
*ω*	Incubation relative rate	days^−1^	0.1429	0.05556–0.5	Analysis of data
*ω*’	Latent relative rate	days^−1^	0.1429	0.05556–0.5	Analysis of data
*γ*	Recovered/Removed rate of the infectious	days^−1^	0.2	0.1111–0.3333	Analysis of data
*γ*’	Recovered/Removed rate of the asymptomatic	days^−1^	0.2	0.04762–1	Analysis of data
*f*	Fatality of the disease	1	0.003552	0–1	Analysis of data

^a^: *i* and *j* represent age group 1 to 4, respectively, and *i* ≠ *j*; - means not applicable

### Quantification of age-specific transmissibility of SARS-CoV-2

The age-specific secondary attack rate (SAR) matrix is defined as the four-by-four matrix with elements *SAR*_*ij*_ = *β*_*ij*_/*γ*, i.e. the rate per encounter at which the virus spreads from age group *i* to age group *j* divided by the frequency of removal. The diagonal elements of the matrix give the age-specific *SAR* values within each age group. Instead of the directly calculated *SAR* value, the min-max normalized (the lower and upper bounds of relative transmissibility) version is used:

$$ \mathrm{Normalized}=\frac{x-\min (x)}{\max (x)-\min (x)} $$

Moreover, a “knock-out” simulation was performed as in reference [29] to quantify the age-specific transmissibility of SARS-CoV-2. To “knock out” means to cut off the transmission route between or within the various age groups. The simulation was performed for the following scenarios: A) *β*_*ii*_ = 0; B) *β*_*ji*_ = 0; C) *β*_*ij*_ = 0; D) *β*_*jj*_ = 0; and E) control (no cutting off of the transmission route).

### Simulation method and statistical analysis

Berkeley Madonna 8.3.18 (developed by Robert Macey and George Oster of the University of California at Berkeley; Copyright© 1993–2001 Robert I. Macey & George F. Oster, University of California, Berkeley, USA) was employed to perform the curve fitting and simulation. The simulation methods (Runge–Kutta method of order four with tolerance set to 0.001) were the same as those used in previously published research [30–36]. Berkeley Madonna adopted the curve fitting of the least root-mean-square deviation. The data were analyzed using Microsoft Office Excel 2016 (Microsoft, Redmond, USA) and GraphPad Prism 7.0 (GraphPad Software, La Jolla, USA). Analyses of the incubation period (using the gamma, normal, lognormal, skew-normal, log-gamma, Weibull minimum, chi-square, Wald, Laplace, and exponential distributions) and the days from illness onset to diagnosis (chi-square distribution) were performed using Python software, version 3.6.1 (Copyright© 2001–2017; Python Software Foundation, Powered by Heroku). The goodness of fit was judged by the coefficient of determination (*R*^2^) value, calculated using SPSS 21.0 (IBM Corp, Armonk, USA).

### Sensitivity analysis

In this study, six parameters were used to analyze the sensitivity of the model: *κ* (0–1), *p* (0.016–0.78), *ω* (0.05556–0.5), *γ* (0.1111–0.3333) and *γ*’ (0.04762–1), each split into 1000 values according to its range. The values of the mean and standard deviation (*SD*) were calculated for the sensitivity analysis.

## Results

### Epidemiological characteristics and of COVID-19

**Fig. 5:**
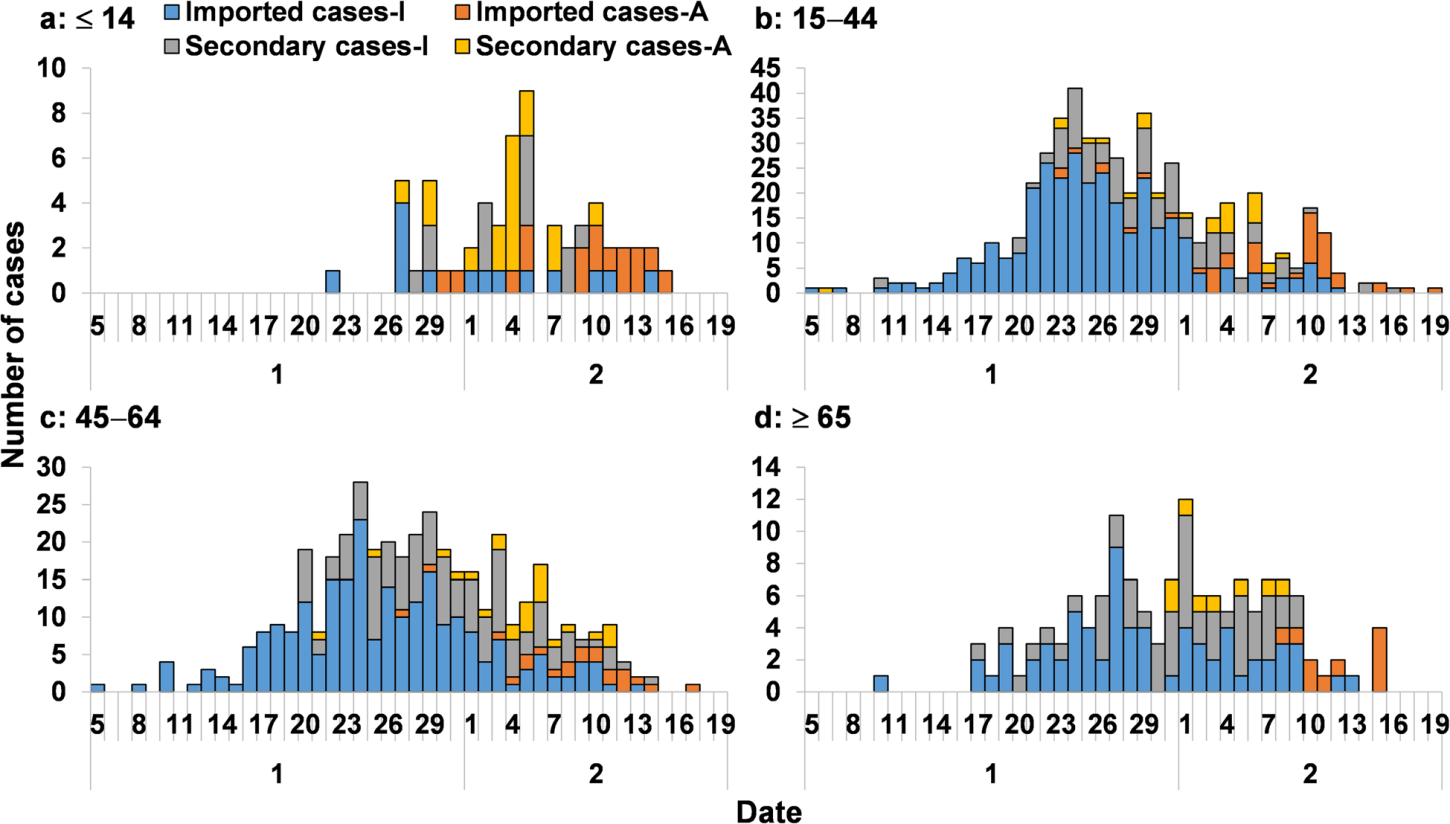
Epidemic curve of reported COVID-19 cases in Hunan Province from January 5 to February 19, 2020. **a** ≤ 14 years; **b** 15–44 years; **c** 45–64 years; **d** ≥ 65 years

Data for 1126 COVID-19 cases were collected in Hunan Province from January 5 to February 19, 2020. The data showed that 734 cases involved people with a history of traveling to other provinces and 392 were secondary cases in Hunan Province (Fig. 5). In the ≤ 14 years old group, there were 14 imported symptomatic cases, 16 imported asymptomatic cases, 13 secondary symptomatic cases, and 17 secondary asymptomatic cases. In the 15 to 44 years old group, there were 318 imported symptomatic cases, 51 imported asymptomatic cases, 118 secondary symptomatic cases, and 29 secondary asymptomatic cases. In the 45 to 64 years old group, there were 234 imported symptomatic cases, 22 imported asymptomatic cases, 129 secondary symptomatic cases, and 25 secondary asymptomatic cases. In the ≥ 65 years old group, there were 69 imported symptomatic cases, 10 imported asymptomatic cases, 53 secondary symptomatic cases, and 8 secondary asymptomatic cases.

**Fig. 6:**
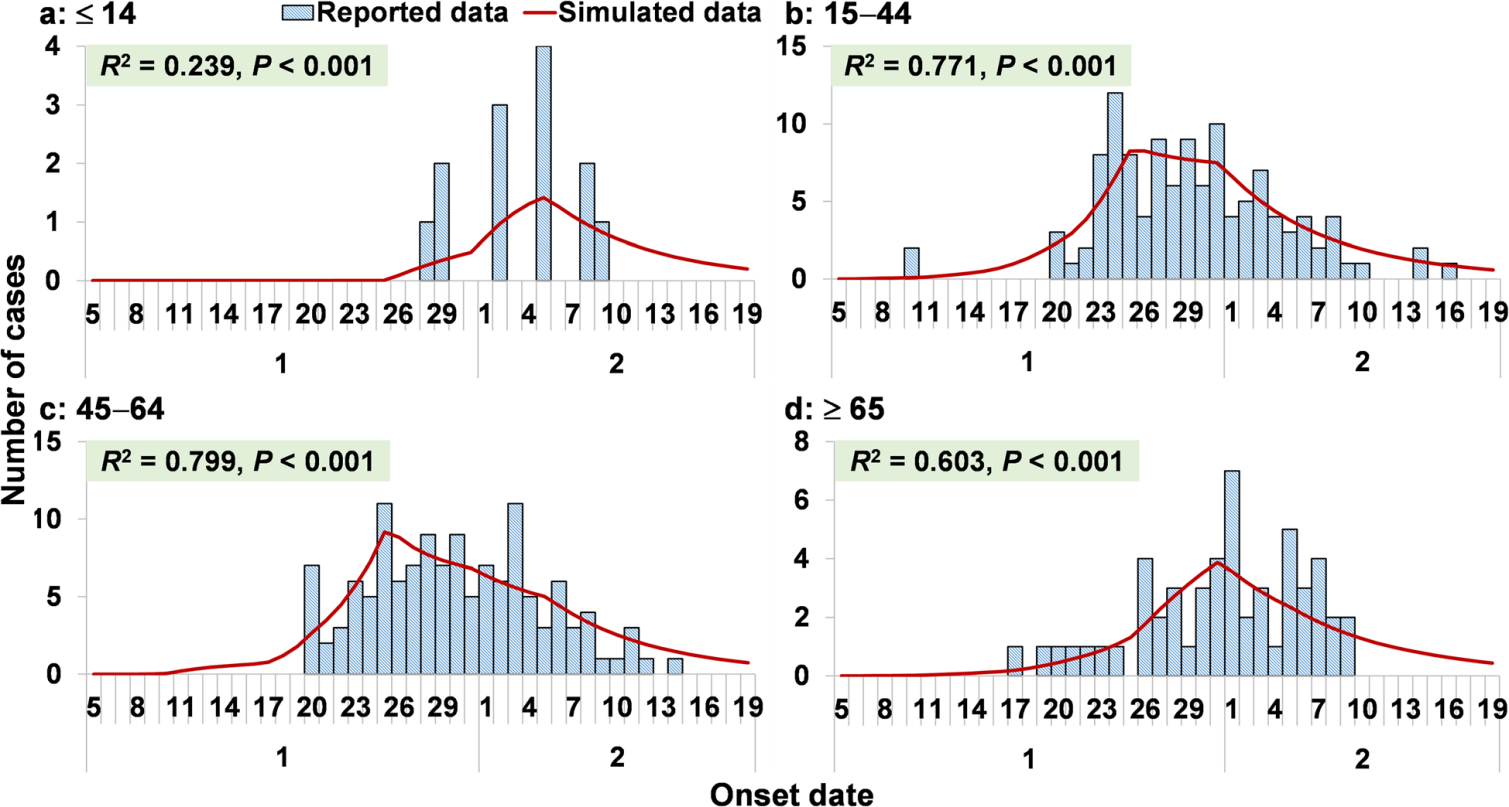
Results of curve fitting of the age-specific SEIAR model to the reported data in Hunan Province. **a** ≤ 14 years; **b** 15–44 years; **c** 45–64 years; **d** ≥ 65 years

**Fig. 7:**
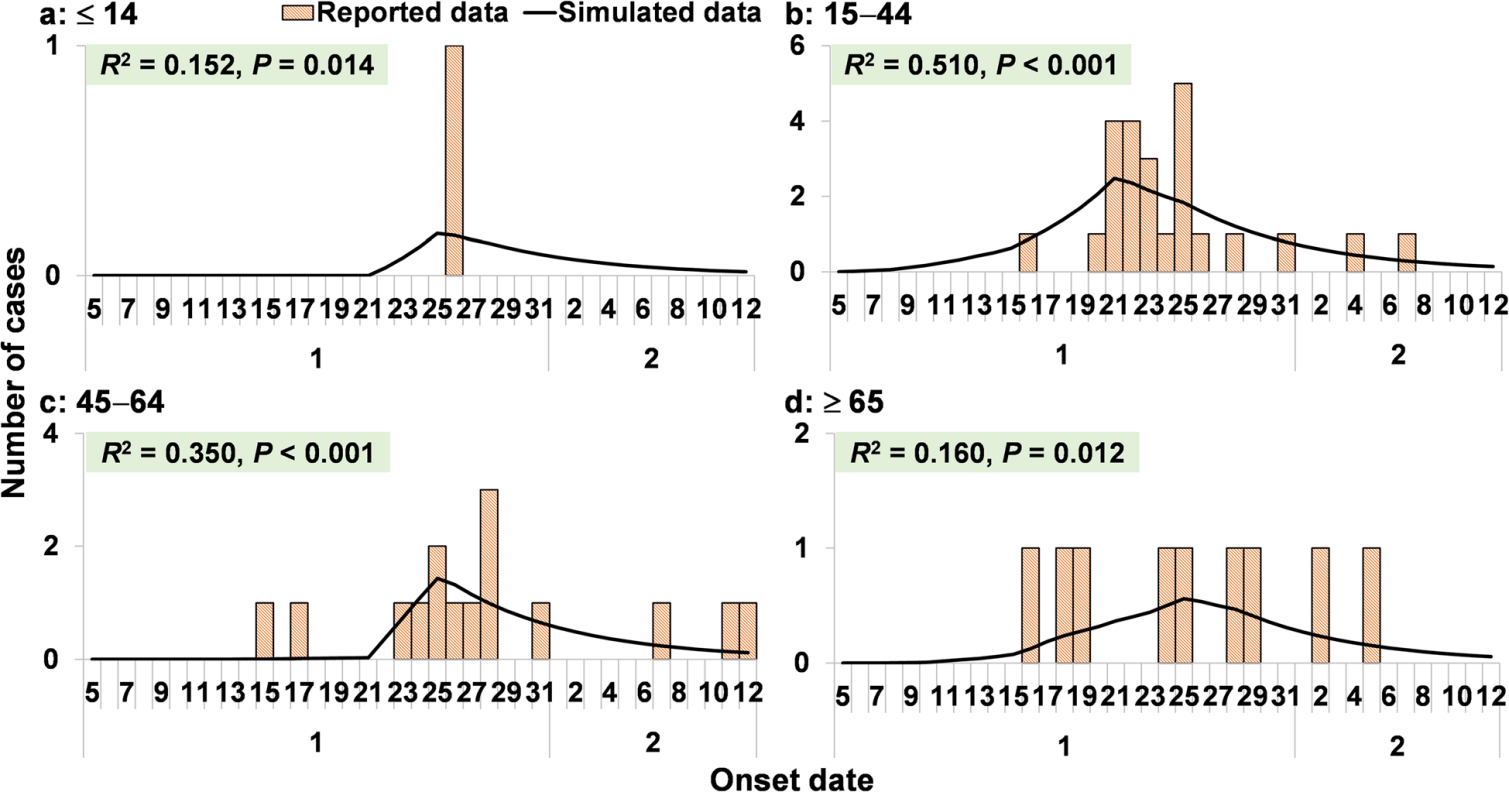
Results of curve fitting of the age-specific SEIAR model to the reported data in Jilin Province. **a** ≤ 14 years; **b** 15–44 years; **c** 45–64 years; **d** ≥ 65 years

In Hunan Province, the age-specific SEIAR model was able to fit the reported data for age group 1 (≤ 14 years: *R*^2^ = 0.239, *P* < 0.001). However, the model fit the reported data for the other three age groups more effectively (15 to 44 years: *R*^2^ = 0.771, *P* < 0.001; 45 to 64 years: *R*^2^ = 0.799, *P* < 0.001; ≥ 65 years: *R*^2^ = 0.603, *P* < 0.001). The results of the curve fitting are displayed in Fig. 6. The age-specific model could also fit the data (Fig. 7) from Jilin Province (≤ 14 years: *R*^2^ = 0.152, *P* = 0.014; 15 to 44 years: *R*^2^ = 0.510, *P* < 0.001; 45 to 64 years: *R*^2^ = 0.350, *P* < 0.001; ≥ 65 years: *R*^2^ = 0.160, *P* = 0.012).

### Transmissibility of SARS-CoV-2

**Fig. 8:**
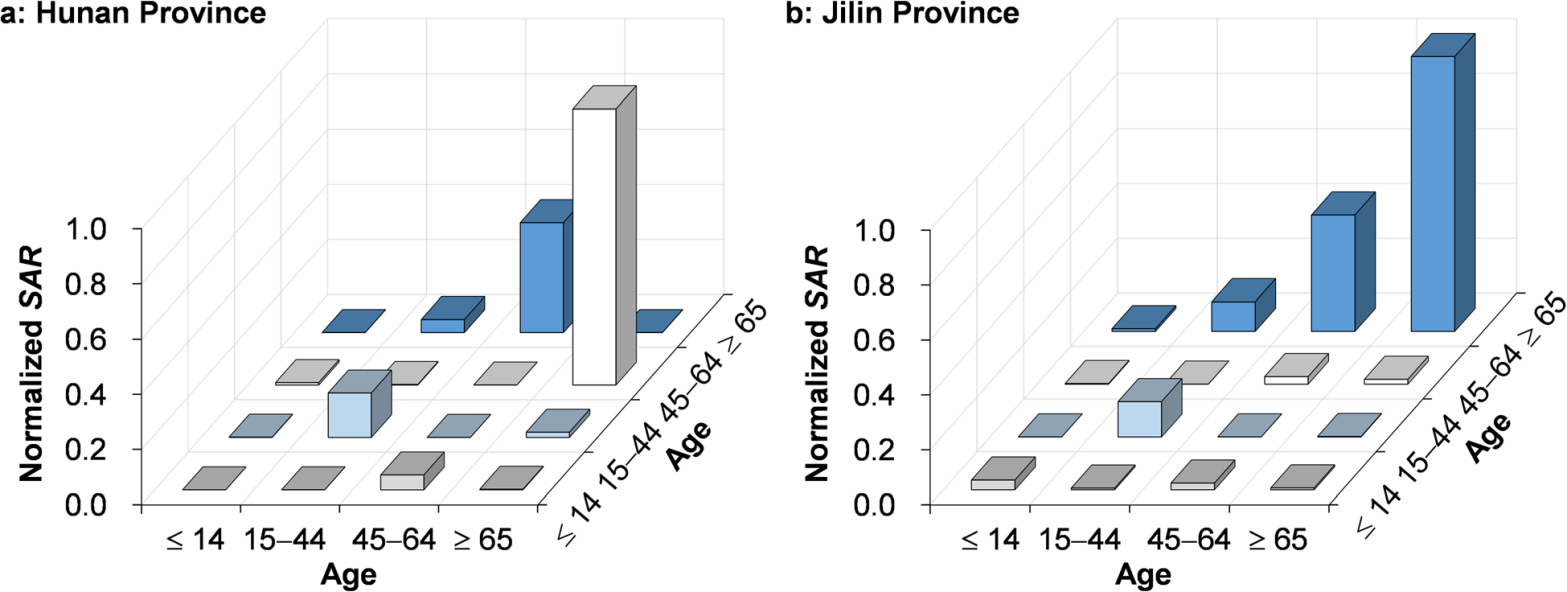
Results of normalized *SAR* value of Hunan and Jilin Province. **a** Hunan province; **b** Jilin province

**Fig. 9:**
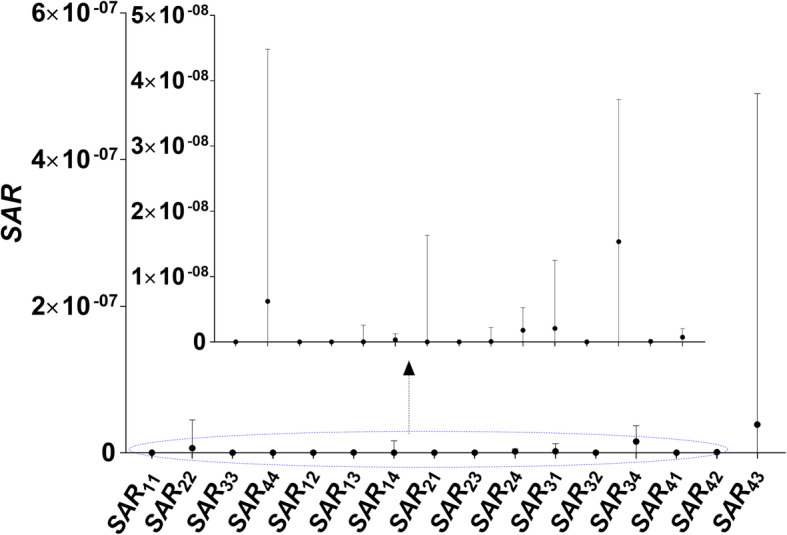
The median and range of *SAR* value in transmission of four age groups of Hunan Province. **1** ≤ 14 years; **2** 15–44 years; **3** 45–64 years; **4** ≥ 65 years

Based on the age-specific SEIAR model, the values of *β*_*ij*_ were consistent with *SAR*_*ij*_ (with *i* and *j* used to represent age groups 1 to 4). In Hunan Province, the highest transmissibility occurred from age group 4 to 3 (median: *β*_43_ = 7.71 × 10^− 9^; *SAR*_43_ = 3.86 × 10^− 8^ [range: 3.03 × 10^− 12^–4.90 × 10^− 7^]), followed by age group 3 to 4 (median: *β*_34_ = 3.07 × 10^− 9^; *SAR*_34_ = 1.53 × 10^− 8^ [range: 4.28 × 10^− 14^–3.71 × 10^− 8^]), age group 2 to 2 (median: *β*_22_ = 1.24 × 10^− 9^; *SAR*_22_ = 6.21 × 10^− 9^ [range: 1.10 × 10^− 11^–4.48 × 10^− 8^]), and age group 3 to 1 (median: *β*_31_ = 4.10 × 10^− 10^; *SAR*_31_ = 2.08 × 10^− 9^ [range: 2.32 × 10^− 14^–1.25 × 10^− 8^]). The lowest transmissibility occurred from age group 3 to 3 (median: *β*_33_ = 1.64 × 10^− 19^; *SAR*_33_ = 8.19 × 10^− 19^ [range: 2.67 × 10^− 19^–2.88 × 10^− 18^]), followed by age group 4 to 4 (median: *β*_44_ = 3.66 × 10^− 17^; *SAR*_44_ = 1.83 × 10^− 16^[range: 1.41 × 10^− 16^–9.07 × 10^− 16^]), age group 3 to 2 (median: *β*_32_ = 1.21 × 10^− 16^; *SAR*_32_ = 6.06 × 10^− 16^ [range: 2.02 × 10^− 19^–2.18 × 10^− 15^]), and age group 1 to 4 (median: *β*_14_ = 7.20 × 10^− 14^; *SAR*_14_ = 3.60 × 10^− 13^ [range: 1.75 × 10^− 13^–1.63 × 10^− 8^]) (Tables 3 and 4). The results for normalized *SAR* are shown in Fig. 8a, and the median and range of *SAR* are displayed in Fig. 9. In Jilin Province (Fig. 8b), the highest transmissibility occurred from age group 4 to 4 (median: *β*_43_ = 4.27 × 10^− 8^; *SAR*_43_ = 2.13 × 10^− 7^ [range: 2.14 × 10^− 10^–7.69 × 10^− 7^]), followed by age group 3 to 4 (median: *β*_34_ = 1.81 × 10^− 8^; *SAR*_34_ = 9.03 × 10^− 8^ [range: 3.03 × 10^− 13^–3.85 × 10^− 7^]). The lowest transmissibility occurred from age group 3 to 2 (median: *β*_33_ = 2.75 × 10^− 14^; *SAR*_33_ = 1.38 × 10^− 13^ [range: 6.91 × 10^− 17^–6.55 × 10^− 10^]), followed by age group 1 to 2 (median: *β*_44_ = 2.19 × 10^− 12^; *SAR*_44_ = 1.09 × 10^− 11^ [range: 1.89 × 10^− 16^–9.46 × 10^− 10^]).

**Table 3:** The median of *β*_*ij*_ (*i* and *j* represent ≤ 14, 15–44, 45–64 and ≥ 65 years old, respectively) calculated by age-specific SEIAR model

Age (years)	≤ 14	15–44	45–64	≥ 65
**≤ 14**	1.70 × 10^−13^	1.12 × 10^− 12^	4.10 × 10^− 10^	1.66 × 10^− 11^
**15–44**	7.16 × 10^− 12^	1.24 × 10^− 9^	1.21 × 10^− 16^	1.45 × 10^− 10^
**45–64**	6.63 × 10^− 11^	1.46 × 10^− 11^	1.64 × 10^− 19^	7.71 × 10^− 9^
**≥ 65**	7.20 × 10^− 14^	3.61 × 10^− 10^	3.07× 10^− 9^	3.66 × 10^− 17^

**Table 4:** The median of *SAR*_*ij*_ (*i* and *j* represent ≤ 14, 15–44, 45–64 and ≥ 65 years old, respectively) calculated by age-specific SEIAR model

Age (years)	≤ 14	15–44	45–64	≥ 65
**≤ 14**	8.50 × 10^−13^	5.59 × 10^− 12^	2.08 × 10^− 9^	8.29 × 10^− 11^
**15–44**	3.58 × 10^− 11^	6.21 × 10^− 9^	6.06 × 10^− 16^	7.27 × 10^− 10^
**45–64**	3.32 × 10^− 10^	7.31 × 10^− 11^	8.19 × 10^− 19^	3.86 × 10^− 8^
**≥ 65**	3.60 × 10^− 13^	1.80 × 10^− 9^	1.53 × 10^− 8^	1.83 × 10^− 16^

**Fig. 10:**
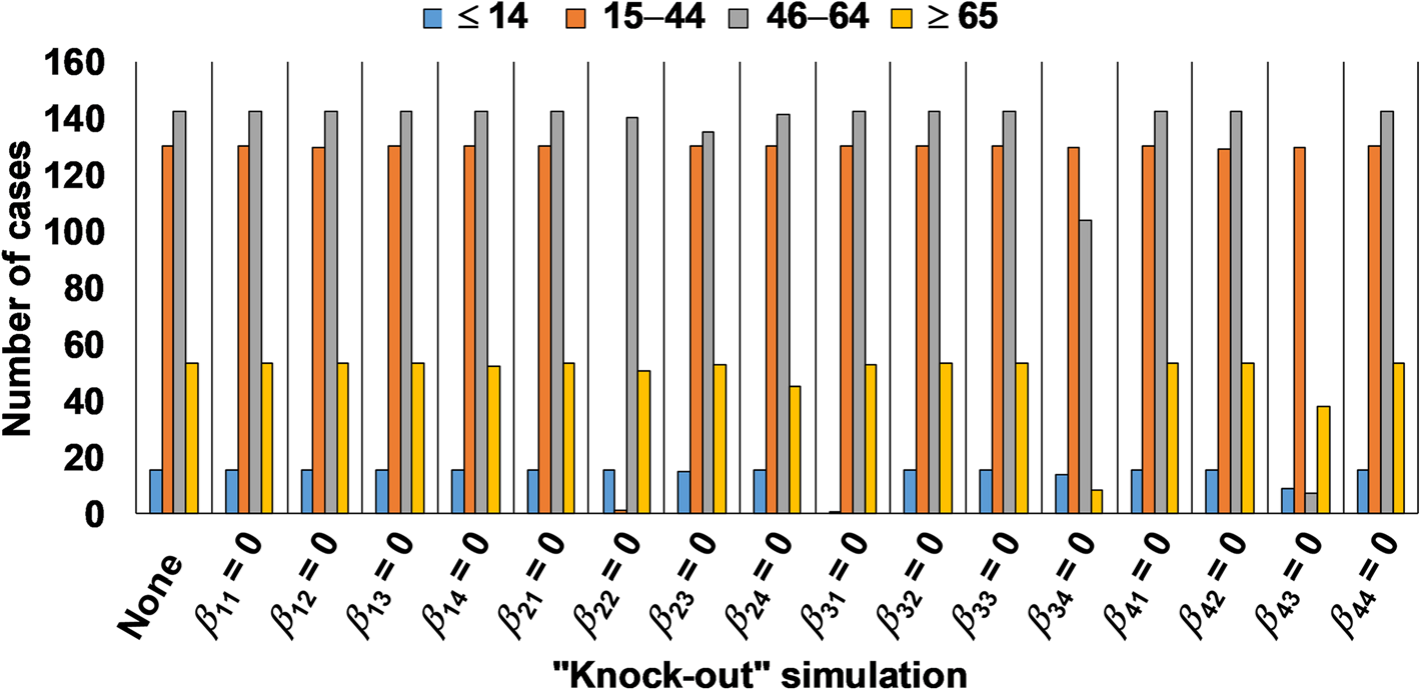
Results of the “knock-out” simulation from the age-specific SEIAR model. *β*_*ij*_ refers to transmission relative rate of age group from *i* to *j*, *i* and *j* represent subscript 1 to 4, subscript 1 was defined as ≤ 14 years, subscript 2 was defined as 15–44 years, subscript 3 was defined as 45–64 years, subscript 4 was defined as ≥ 65 years

The results of the “knock-out” simulation demonstrated that the scenarios *β*_43_ = 0, *β*_22_ = 0, *β*_34_ = 0, *β*_31_ = 0, *β*_24_ = 0, and *β*_23_ = 0 resulted in the highest decrease in the total number of cases (Fig. 10). In age group 1, the results indicated that the scenarios *β*_31_ = 0, *β*_43_ = 0, *β*_34_ = 0, and *β*_23_ = 0 led to the highest decrease. In age group 2, the results demonstrated that the scenarios *β*_22_ = 0 and *β*_42_ = 0 led to the highest decrease. In age group 3, the results indicated that the scenarios *β*_43_ = 0, *β*_34_ = 0, *β*_23_ = 0, and *β*_24_ = 0 led to the highest decrease. In age group 4, the results demonstrated that the scenarios *β*_34_ = 0, *β*_43_ = 0, *β*_24_ = 0, and *β*_22_ = 0 led to the highest decrease.

### Sensitivity analysis

**Fig. 11:**
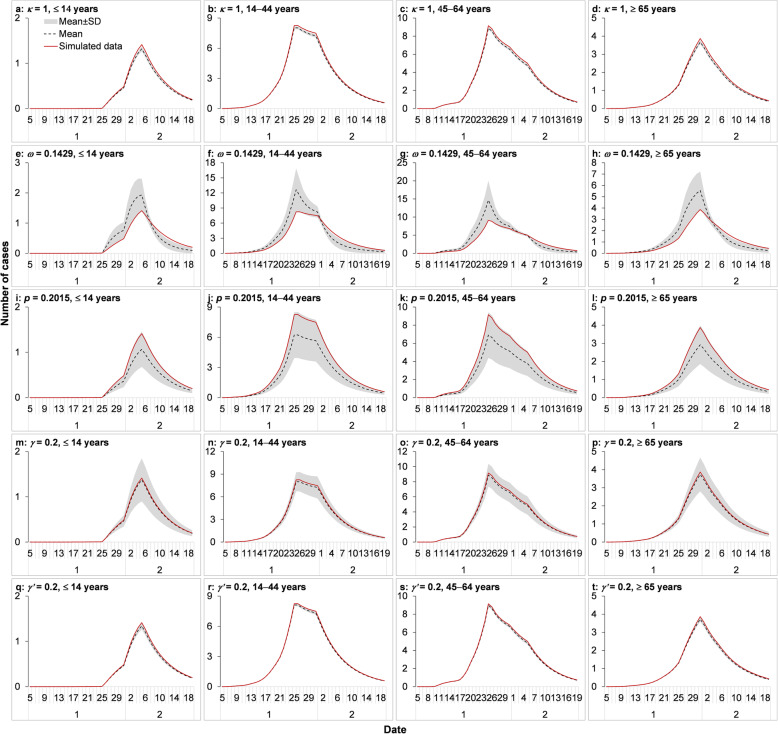
Sensitivity analysis of *κ*, *p*, *ω*, *γ* and *γ*’ parameters in Hunan Province. **a** *κ* = 1, ≤ 14 years; **b** *κ* = 1, 15–44 years; **c** *κ* = 1, 45–64 years; **d** *κ* = 1, ≥ 65 years; **e** *ω* = 0.1429, ≤ 14 years; **f** *ω* = 0.1429, 15–44 years; **g** *ω* = 0.1429, 45–64 years; **h** *ω* = 0.1429, ≥ 65 years; **i** *p* = 0.2015, ≤ 14 years; **j** *p* = 0.2015, 15–44 years; **k** *p* = 0.2015, 45–64 years; **l** *p* = 0.2015, ≥ 65 years; **m** *γ* = 0.2, ≤ 14 years; **n** *γ* = 0.2, 15–44 years; **o** *γ* = 0.2, 45–64 years; **p** *γ* = 0.2, ≥ 65 years; **q** *γ*’ = 0.2, ≤ 14 years; **r** *γ*’ = 0.2, 15–44 years; **s** *γ*’ = 0.2, 45–64 years; **t** *γ*’ = 0.2, ≥ 65 years

In this study, we found that all the values of the parameters we set in the model were included in the range of the simulated values of mean ± *SD*. The three parameters *p*, *ω*, and *γ* were very sensitive for the model, whereas *κ* and *γ*’ were not (Fig. 11).

## Discussion

This is the first study to develop an age-specific SEIAR model for quantifying the transmissibility of COVID-19 within and between various age groups. The model fitted the reported data effectively, and therefore offers the capability of estimating or predicting the age-specific transmissibility of the virus.

For COVID-19, as for other epidemic diseases, the numbers of reported cases differed among people of various ages [2, 18, 37]. One study indicated an especially high rate of infection in 30–79 years age group [3]. This is similar to the present study’s finding that the highest numbers of cases occur among persons 15–44 and 45–64 years old. It is also important to distinguish imported cases from secondary ones. In this study, it was found that susceptible persons in Hunan Province are most often infected by imported cases from elsewhere (especially from Wuhan City). This finding suggested that the monitoring and management of imported cases should be improved further.

The age-specific model fitted the reported data effectively in three age groups, but less effectively for age group 1 (≤ 14 years old). The poor fit in age group 1 was a result of the low number of cases. However, the age-specific SEIAR model was still suitable for this study. These results were consistent with an earlier study on shigellosis using an age–sex-specific SEIAR model [29].

According to the model for Hunan Province, the highest transmissibility occurred from ≥ 65 years to 45–64 years, followed by that from 45 to 64 years to ≥ 65 year-olds, that among 15–44 year-olds, and that from 45 to 64 year-olds to ≤ 14 year-olds. Another study, adopting a generalized linear mixed model to divide the total population into three age groups, found that the risk of infection in persons ≥ 65 years old is higher than that in persons of 15–64 years old (odds ratio: 1.47, 95% *CI*: 1.12–1.92) [8]. This result differs slightly from that of the present study, perhaps simply as a result of the difference in the manner in which the total population was divided into groups. Note that the generalized linear mixed model cannot be used to assess transmission features in the population, such as influence of asymptomatic and imported cases.

In the model given here, the lowest transmissibility occurred among 45–64 years age group, followed by that among ≥ 65 year-olds, that from 45 to 64 year-olds to 15–44 year-olds, and that from ≤ 14 year-olds to ≥ 65 year-olds. The “knock-out” simulation results differed from the *SAR* values, with the following order: from ≥ 65 year-olds to 45–64 year-olds, among 15–44 year-olds, from 45 to 64 year-olds to ≥ 65 year-olds, and from 45 to 64 year-olds to ≤14 year-olds. According to the model, the virus was most likely to be transmitted between elderly (≥ 65 years old) and middle-aged (45 to 64 years old) people, whereas persons aged 15 to 44 have relatively low susceptibility to COVID-19. This may relate to the custom of middle-aged people caring for their ill parents, resulting in a high contact frequency with the elderly. Although physical fitness and resistance in elders are lower than in younger adults, this study found a high transmissibility from ≥ 65 year-olds to 45–64 year-olds in Hunan Province. This may relate to the lifestyle differences between the generations and to clustering in families. A more detailed study and a larger sample are needed to test the model more extensively. Moreover, relatively high transmissibility was observed among the group comprising 15 to 44 year-olds. This is similar to the age-specific transmissibility of influenza A (H1N1) [13]. Therefore, age-specific control and prevention interventions are necessary. The *SAR* value is very small (nearly zero) because the model was built on the total population of Hunan Province (68 988 303 persons are so big that it drowns out the signal). *SAR* was only used in the comparison of relative transmissibility between different age groups, as in a similar study of a sex-based and age-based model of shigellosis in Hubei Province [29]. The results for Jilin Province (especially the importance of transmission among the elderly) differed somewhat from those for Hunan Province (where the most important transmission route is from middle-aged to elders). This may relate to the small sample size of COVID-19 in Jilin Province. Nevertheless, the most significant transmission in Jilin did involve middle-aged and elderly individuals. Here, too, those 15–44 years old have relatively low susceptibility and those ≤ 14 years old very low susceptibility to COVID-19.

The reasons for the age-specific transmissibility differences remain unclear but may be related to the different kinds of contact characteristics of various age groups. Adults are more likely to work outside and to come into contact with different individuals in workplaces, buses, subways, or airplanes. Even under the powerful intervention and management implemented in China, young and middle-aged people would still engage in certain cluster activities such as visiting relatives and having parties. However, children or younger people may have stayed at home constantly during the outbreak and been less likely to be infected, except by adults or elderly people in the same family.

The results were more certain for all parameters that were collected from first-hand data of Hunan Province. Some studies have indicated that infection may occur at the end of an incubation period [38, 39]. According to one survey, in 59 out of 468 reports the infected person exhibited symptoms earlier than the person who infected them [40], implying that it is possible to infect other people during the incubation period. However, according to the survey conducted by Hunan Provincial CDC, there is no obvious evidence that exposed persons are infective during the incubation period. This issue will have to be resolved for effective application of this model in other areas. Bai et al. [41] reported an asymptomatic proportion of 0.17. Previous research demonstrated that an asymptomatic infection can shed SARS-CoV-2 for 5 days [42]. This is consistent with the parameter values in the proposed model. However, not enough evidence or first-hand data analyses were available to provide clear epidemiological estimates of the parameters *ω*’ and *γ*’, which are related to asymptomatic individuals. Additional epidemiological data are required to explore these parameters. Furthermore, the results of sensitivity analysis also showed that additional accurate first-hand data are needed to better determine the three parameters *p*, *ω*, and *γ*.

Owing to the poor fit in the youngest age group, additional first-hand data are necessary to verify the age-specific SEIAR model. Xie et al. found a positive linear relationship between mean temperature and the number of COVID-19 cases with a threshold of 3 °C [43]. Ma et al. found that the temperature and humidity may affect COVID-19 mortality [44]. Furthermore, some studies have suggested that COVID-19 incidence is connected not only to meteorological factors but also to population size [45–47]. The present study focused on a short period; therefore, these factors were not considered owing to the limited availability of data. In future work, the meteorological factor related to COVID-19 should be further explored.

## Conclusions

The study models the transmission of COVID-19 by and among persons of different ages. The model demonstrates that SARS-CoV-2 exhibits high transmissibility between elderly (≥ 65 years old) and middle-aged (45–64 years old) people. Persons aged 15–44 years have relative low susceptibility to COVID-19 and those aged 14 or less have even lower susceptibility. The majority of prevention measures should be applied to eliminate person-to-person transmission among middle-aged and elderly people.

## Supplementary information

**Additional file 1: Table S1.** The epidemic data of COVID-19 in Hunan Province

## Data Availability

Not applicable.

## Abbreviations

COVID-19: Coronavirus disease 2019
SARS-CoV-2: Severe acute respiratory syndrome coronavirus 2
*R*_0_: Basic reproduction number
*CI*: Confidence interval
BHRP: Bats-Hosts-Reservoir-People
PP: Person-Person
SEIAR: Susceptible – exposed – symptomatic – asymptomatic – recovered/removed
CDC: Center for Disease Control and Prevention
SAR: Secondary attack rate
*R*^2^: Coefficient of determination
*SD*: Standard deviation
